# MRI rectal cancer in Australia and New Zealand: an audit from the PETACC-6 trial

**DOI:** 10.1186/1470-7330-15-S1-P44

**Published:** 2015-10-02

**Authors:** KLM Gormly, C Coscia, T Wells, N Tebbutt, JA Harvey, K Wilson, H-J Schmoll, T Price

**Affiliations:** 1 Dr Jones and Partners Medical Imaging, Adelaide, Australia; 2 Olivia Newton-John Cancer and Wellness Centre, Austin Health. Melbourne, Melbourne, Australia; 3 Princess Alexandra Hospital, Woolloongabba, Australia; 4 NHMRC Clinical Trials Centre, University of Sydney, Sydney, Australia; 5 Martin Luther University, Halle, Germany; 6 The Queen Elizabeth Hospital, University of Adelaide, Adelaide, Australia; 7 AGITG, Australia

## Introduction

An MRI audit substudy was conducted of patients who underwent an MRI prior to treatment in Australia and New Zealand as part of the international PETACC-6 trial in locally advanced rectal cancer.

## Method

82 patients of the 127 Australasian patients from 15 centres had rectal MRI scans reviewed for technique, data included in reports and comparison of reports with blinded central reporting by 2 experienced radiologists.

## Results

82% performed minimum T2 sagittal and T2 axial oblique sequences. The high resolution T2 sequence parameters varied significantly with only 33% obtaining a voxel size of ≤1.3. The rate of inclusion of relevant findings in the reports was; T3 distance in mm 21%, N stage 84%, CRM status 72%, EMVI status 29% and distance from the puborectalis sling 17%. 31% of reports included all of; T stage with T3 substage, N stage and CRM involvement. 17% of reports included these 3 findings and EMVI. Eleven reports used a template with 82% of these including the first 3 findings. The agreement with central reporters was T stage 76%, N stage 70%, CRM status 57% and EMVI 16%.

## Conclusion

There is significant variation in scan quality and low rates of inclusion of all clinically relevant findings in rectal MRI reports reviewed for this audit. The authors recommend adoption of routine sequences and template reports to improve scan technique and report accuracy in rectal cancer MRI staging scans across Australia and New Zealand.

